# “This is my boy’s health! Talk straight to me!” perspectives on accessible and culturally safe care among Aboriginal and Torres Strait Islander patients of clinical genetics services

**DOI:** 10.1186/s12939-021-01443-0

**Published:** 2021-04-17

**Authors:** Philippa Dalach, Ravi Savarirayan, Gareth Baynam, Julie McGaughran, Emma Kowal, Libby Massey, Misty Jenkins, Yin Paradies, Margaret Kelaher

**Affiliations:** 1 Centre for Health Policy, School of Population and Global Health, University of Melbourne, Parkville, Victoria Australia; 2 Victorian Clinical Genetics Services, Murdoch Children’s Research Institute and University of Melbourne, Parkville, Victoria Australia; 3 Western Australian Department of Health, Genetic Services of Western Australia, Perth, Western Australia Australia; 4 Western Australian Register of Developmental Anomalies, Western Australian Department of Health, Perth, Australia; 5 Telethon Kids Institute and Division of Paediatrics, Faculty of Health and Medical Sciences, University of Western Australia, Perth, Australia; 6 Genetic Health Queensland, Royal Brisbane & Women’s Hospital, Brisbane, Queensland Australia; 7 School of Medicine, University of Queensland, St Lucia, Queensland Australia; 8 Alfred Deakin Institute for Citizenship and Globalisation, Deakin University, Geelong, Victoria Australia; 9 Machado Joseph Disease Foundation, Alyangula, Northern Territory Australia; 10 James Cook University, Townsville, Queensland Australia; 11 Walter & Eliza Hall Institute of Medical Research, Parkville, Victoria Australia

**Keywords:** Indigenous Australians, Aboriginal and Torres Strait islanders, Genetic health services, Access to health care, Cultural safety

## Abstract

**Background:**

Aboriginal and Torres Strait Islander people do not enjoy equal access to specialist health services that adequately meet their needs. Clinical genetics services are at the vanguard of realising the health benefits of genomic medicine. As the field continues to expand in clinical utility and implementation, it is critical that Aboriginal and Torres Strait Islander people are able to participate and benefit equally to avoid further widening of the existing health gap. This is the first study to explore barriers to accessing clinical genetics services among Aboriginal and Torres Strait Islander people, which has been acknowledged as a key strategic priority in Australian genomic health policy.

**Methods:**

A participatory design process engaged a majority-Aboriginal Project Reference Group and Aboriginal End-User Group. 63 semi-structured interviews were conducted with Aboriginal and/or Torres Strait Islander people who had accessed the government-funded clinical genetics service in Western Australia, Queensland or the Northern Territory between 2014 and 2018. The sample included patients, parents and carers. Participants were asked to recount their ‘patient journey’, from referral through to post-appointment and reflect on their perceptions of genetics and its implications for the health of themselves and their families. Analysis tracked chronological service engagement, followed by an inductive thematic approach.

**Results:**

Barriers to access and engagement were present at each stage of the patient journey. These included challenges in obtaining a referral, long waiting periods, limited genetic literacy, absence of Aboriginal support services, communication challenges and lack of adequate psychosocial support and follow-up after attendance. Participants’ overall experiences of attending a genetic health service were varied, with positive perceptions tied closely to a diagnosis being achieved. The experience of (and expectation for) recognition of cultural identity and provision of culturally safe care was low among participants. Unaddressed concerns continued to cause significant distress in some people years after their appointment took place.

**Conclusions:**

There is significant scope for improving the care provided to Aboriginal and Torres Strait Islander people at clinical genetics services. Immediate attention to minimising logistical barriers, developing relationships with Aboriginal Community Controlled Health Services and providing practical and specific cultural safety training for practitioners is required at the service-level. Our findings strongly support the development of guidelines or policies recognising the collective cultural needs of Aboriginal and Torres Strait Islander people in relation to genomic health care.

**Supplementary Information:**

The online version contains supplementary material available at 10.1186/s12939-021-01443-0.

## Background

There has been considerable progress achieved in the past decade in the understanding, diagnosis, treatment and prevention of heritable and other genetically determined conditions. The advent of affordable and accessible genomic sequencing technologies has significantly improved the clinical management of some patients through improved diagnosis and access to targeted treatments [1], most notably in the fields of oncology [2], rare diseases [3] and personalised pharmacology [4]. Clinical genetics services are specialist services that encompass a broad range of activities and capabilities, with functions relevant to patients throughout the life course, from pre-implantation genetic diagnosis to prenatal, paediatric and adult genetics [5, 6] and are at the vanguard of translating recent advances into better patient outcomes. Some of the distinct challenges faced by clinical genetic services will ultimately impact the health system as a whole, as the availability and utility of genomic information increases. Equitable service delivery in clinical genetics is therefore critical to serving the most and the most in need, now and into the future.

The importance of the equitable development of genomic medicine has been recognised internationally [7] and in Australia [8]. However to date most of the published focus has been on inclusion in research (e.g. [9, 10]) particularly against the backdrop of unethical and harmful in genetic research on Aboriginal and Torres Strait Islander people in the past [11]. There is an emerging literature on the importance of developing genomic reference data specific to Aboriginal and Torres Strait Islander people to assist in the diagnosis of genomic conditions for these populations [12, 13]. Lack of appropriate reference data has led to delays in the diagnosis of genetic/genomic conditions and the use of suboptimal treatments among non-Europeans [14]. Harmful delays in diagnosis and adverse drug reactions due to the use of medicines that were unsafe based on a patient’s pharmacogenetic profile have been recognised among Aboriginal and Torres Strait Islander people [15]. However at time of writing there has been no studies on disparities in access to genomic medicine among Aboriginal and Torres Strait Islander people [16]. This in turn has meant that discourse around genomic medicine has focussed on future benefit rather than the more tangible and imperative benefits of ensuring Aboriginal and Torres Strait Islander people have access to the services they require now.

The United Nations Declaration on the Rights of Indigenous Peoples [17] encompasses obligation to ensure equity in genomic health for Aboriginal and Torres Strait Islander people as Australia’s first people and a population unique to Australia. In Australia, the strategic priorities of the National Health Genomics Policy Framework 2018–2021 [18] include the need to ‘[i]dentify barriers to equity of access and develop a national approach to address these, noting that access is multi-dimensional and includes location, cost, availability and appropriateness (including cultural acceptability).’ Current equity standards in clinical genetics typically only include monitoring referral rates by region [19],^1^ and this is inconsistently implemented by services. There is no focus on standards or training to ensure that quality of care is consistent across a diverse range of clients to improve equity within clinical interactions [6]. It is imperative to developing the evidence base to develop better standards for clinical practice and training for inclusion in the next iteration of the National Health Genomics Policy Framework.

State and Territory clinical genetics services in Australia are usually hospital based and delivered through a state-wide hub and spoke model service. An exception is the Northern Territory where services are operated on a fly-in fly-out basis. Clinical genetic services accept inpatient and outpatient referrals. Services are provided without cost to patients. Patient interactions with the services are focussed on the provision of genetic diagnoses and test results. The physical and mental health sequalae of these results are generally managed by other specialist services. There is a small amount of literature from Australia looking at patients’ experiences of accessing clinical genetics services. Among 397 attendees of a Victorian clinical genetics service who responded to a mail questionnaire, overall satisfaction with their experience was very high (82.4% satisfied or very satisfied) [20]. It should be noted that people who ‘were from a non-English speaking backgrounds’ were excluded from this study. Satisfaction has also been shown to be high in the context of a familial colorectal cancer clinic, where attendance was also shown to alleviate worry for many people [21], and among families of children diagnosed with a rare disease accessing a specialised genetic service, where 40% of participants indicated that they experienced no barriers to access at all [22]. People who were dissatisfied with the of experience of receiving a diagnosis in the rare disease study (13%) was most commonly attributed to poor communication style and inadequate provision of appropriate information and psychosocial support [22].

Aboriginal and Torres Strait Islander people make up 3.3% of the Australian population and represent hundreds of distinct cultural and linguistic groups [23]. Most (81%) live in non-remote areas of Australia, however the proportion of the population that is Aboriginal and Torres Strait Islander is much higher in remote (18%) and very remote (47%) regions compared to non-remote regions (2.7%). The Aboriginal and Torres Strait Islander population also has a younger age structure than the rest of the Australian population, with a lower median age (23.0 vs 37.8 years), higher proportion aged under 15 years (34% vs 18%) and lower proportion over the age of 65 (4% vs 16%). This in part relates to the significantly higher mortality rates among Aboriginal and Torres Strait Islander people in the 35–44 years age group, relative to other Australians [24]. Importantly, the Aboriginal and Torres Strait Islander population has a higher incidence of cancer and lower survival rates than other Australians [25]. It is likely that there is a hereditary component to the aetiology in a proportion of these cases of cancer, where attendance at a clinical genetics service may benefit the patient and other family members.

Aboriginal and Torres Strait Islander people are disadvantaged in terms of access to specialist care, and clinical genetic services are no exception [26, 27]. Preliminary data from the Northern Territory Genetic Service suggested that rates of referral for Aboriginal and Torres Strait Islander patients were less than half of what would be expected based on population estimates [28]. This is particularly concerning given that Aboriginal and Torres Strait Islander people have a higher incidence of some inherited conditions [26], most notably cancer [29]. There is also evidence for a desire to be included in genomic health: a study by Bernades et al. [30] found that among 252 Aboriginal and Torres Strait Islander cancer patients in Queensland, nearly three-quarters (73%) of had a family history of cancer, 68.3% were concerned about the potential risk of cancer for family members, and over half (54.4%) were interested in talking to a specialist about the implications of their diagnosis for their relatives. Finally, there is limited existing capacity and training to provide high-quality care to Aboriginal and Torres Strait Islander patients. Kowal et al. [6] found that genetic counsellors espouse a patient-centred approach which does not readily allow for consideration of collective cultural needs. This also has important implications for other minority patient groups. The need for culturally tailored approaches to genetic counselling or inclusion in genomic research has been explored in First Nations populations in Aotearoa New Zealand [31], Canada [32], a migrant Indigenous population from Oaxaca living in the Central Valley of California [33] and other ‘diverse underserved’ patient populations in the United States [34]. These small, exploratory studies all found that incorporating cultural beliefs into genetic counselling was essential in order to achieve acceptable outcomes for patients. These findings have been echoed in community co-designed genetic services in Australia which also highlight the importance of gender in developing culturally appropriate services [35].

In this paper, we examine the experiences of Aboriginal and Torres Strait Islander people who had attended a mainstream clinical genetics service in the past (as a patient or carer). By exploring their perceptions of barriers and enablers to attending, and the quality of care provided to them, we have developed recommendations for readily implementable changes to improve quality of care throughout the patient journey to and from clinical genetics services.

## Methods

### Study design

To better understand equity of access and perceptions of the clinical experience among Aboriginal and Torres strait Islander people, we conducted semi-structured interviews with people who had attended a publicly funded genetics service in three Australian states/territories (Western Australia, Queensland and the Northern Territory).

Interview protocols were designed based on Penchansky and Thomas’s model of access to health care [36] and further developed and refined in collaboration with a Project Reference Group and End-User Group. The Project Reference Group was made up of government policy makers, academic and clinical experts in genetics and Indigenous health (Australian and international; 11 of 25 members incuding the research team identifying as Aboriginal and Torres Strait Islander and an additional 4 members identifying as First Nations people from other countries) and the EUG comprised seven Aboriginal women from across Australia, who had personal experience in accessing a clinical genetics service and/or working in Aboriginal health or community services.

Ethics approval was obtained from the following Human Research Ethics Committees: The University of Melbourne (HREC-1648489.4), Northern Territory Department of Health and Menzies School of Health Research (HREC-2018-3075) and the Central Australian Health Service (HREC-18-3112), The Queensland Department of Health (HREC/18/QTHS/51), the Aboriginal Health Council of Western Australia (HREC-810) and the King Edward Memorial Hospital (RGS0000000513). Additionally, support from the project was received from relevant local Aboriginal Health Organisations as part of extensive stakeholder consultation and engagement activities.

### Aboriginal and Torres Strait islander patients

Interviews with Aboriginal and Torres Strait Islander patients of clinical genetics services allowed participants to discuss their experiences of attending a clinical genetics service by recounting their journey before, during and after their appointments and having a conversation about this with the interviewer (see Supplementary Document 2 for interview protocol).

Patients were retrospectively recruited from each of the services. Invitations to eligible participants (i.e. patients who had identified themselves as Aboriginal and/or Torres Strait Islander, and who had attended an appointment in 2014–2018) were sent by post, and included a plain language statement describing the research, a consent form and contact details for the research team (post, email and phone/text message) to either opt in or out of the study. Potential participants who had not responded three weeks after the invitation was sent were followed up by phone and invited to participate. A total of 63 interviews were undertaken with patients, parents and carers (Table 1). Due to the rare nature of some genetic conditions and the small number of Aboriginal and Torres Strait Islander people who had accessed the services, detailed demographic information and reason for attending the service was not collected from interviewees in order to ensure that they felt that their anonymity would be preserved. In total only four men were interviewed, all of whom were a parent or guardian of a child being investigated in relation to a rare disease.

**Table 1:** Role and location of interview participants

	Service Location		
Reason for attending, n (%)	QLD	NT	WA
Cancer	8 (24)	4 (21)	0 (0)
Rare disease	25 (74)	14 (74)	10 (100)
Prenatal	1 (3)	1 (5)	0 (0)

Local Aboriginal women were recruited as interviewers in Queensland and Western Australia. In the Northern Territory, a researcher with over 20 years’ experience working in Aboriginal health settings conducted the interviews. All interviewers were trained to administer the interview protocol, including the importance of using open-ended questions, and being flexible to following the participant’s responses and re-wording questions to ensure mutual understanding, as appropriate. Training also included guidance on caring for all participants during the interview process and how to support any person who became distressed or indicated unresolved concerns about their interaction with a health service. Interviewers were supported throughout the data collection by the research team, who provided regular opportunities for debriefing discussions. Participants had the option of completing the interview by phone or in person. Participants were reimbursed for their time with a $50 grocery voucher. Reimbursing Indigenous participants for their time was a pre-requisite for ethics approval. The level of reimbursement was kept consistent with other studies to avoid any inappropriate influence on participation.

### Data analysis

Interviews were audio-recorded and transcribed using Rev Transcription Services [37]. Transcripts were cross-checked by the research team to ensure integrity of the data. NVivo software was used for data management and thematic analysis. There were no consistent differences in the interviews completed in person to the interviews completed over the phone.

Transcripts were coded by a single researcher (PD), initially using the chronological patient journey as a framework to identify key barriers and enablers to service attendance, and then using an inductive approach to identify common, overarching themes across participants. Review of the data and iterative thematic analysis was discussed regularly with the principal researcher (MK). The approach to developing the coding framework and emergent themes were also discussed with the End User Group and Project Reference Group. This reflexive approach ensured that the analysis both captured the experiences of patients and informed an appropriate health system responsive. It is a key principle in Aboriginal and Torres Strait islander research that the key to improving health services for Aboriginal and Torres Strait Islander people requires understanding how services are working from their perspective and incorporating this in the design and governance of services [38]. The governance and analytic approach of the project is designed to privilege Aboriginal and Torres Strait Islander perspectives in all aspects of the research.

## Results

The semi-structured interviews began with the participant recounting the story of attending the clinical genetics in their own words. The following results are presented to align chronologically with the stages of a typical patient journey, with emergent themes highlighted for each stage.

### Pre-appointment

#### Referral process and preparation for attending the service

Three key themes emerged relating to the lead-up to an appointment with genetic services. Firstly, the referral to the clinical genetic service was often one of a large number of referrals received by patients, particularly in the case of referrals for complex paediatric conditions.

*We were pretty much living in and out of the hospital at the time. A lot of everything else, appointments and therapies and just dealing with the prospect of a challenging life to be from this point on. Parent, NT*

Consequently, patients were often unsure about why they had been referred to the genetic service and did not really understand how this appointment related to other healthcare services they were currently accessing. For example, patients were often confused and sometimes anxious about why they were asked to bring all their children to an appointment when, from their perspective, only one child had an issue that needed to be explored. Furthermore, the long wait times experienced compounded confusion about the reason for the appointment. Some parents reported that they had forgotten why they had been referred by the time they were called to the appointment, sometimes over a year after the initial referral.

The second theme was the difficulty in obtaining a referral to a genetic health service. Some patients experienced racism and stigma throughout the process, blocking their access to appropriate care. While this is outside the remit of the clinical genetic services themselves, it does highlight the critical need to ensure culturally safety within clinical genetics services, as well as for boarder systemic change.

*We found the other two fractures and I started obviously asking questions to why he would being having fractures. [The hospital], they had their opinion that it was something that me and my partner did and so they weren't really willing to look past anything else other than that. Parent, NT*

One patient who had a family history of a rare disease recounted having her request for a referral blocked by a primary care doctor who “made [her] feel really silly for wanting it, and saying that … I can’t get an appointment because I don’t have a direct link, when I clearly do.”

The third theme was that patients often arrived at the appointment with a limited understanding of the nature of the appointment, the kinds of questions they might be asked and decisions they might need to make. These issues were exacerbated by the fact that information provided by referring specialist physicians was of variable quality.

*[I] was very confused, yeah, because she didn't quite say that there was something wrong, but she didn't explain to me what [geneticist’s name] was. She didn't really explain anything.*

*So she didn't tell you that it was a genetic doctor?*

*No. No. And she didn't say that I could possibly … we just want to look in to mainly that if he has any genetic growth problems. She didn't even explain that. She just said, "Oh, I want you to see the specialist. I'll book that in for you," and then you get in your car again, then it's just what was that all about? Yeah, Yeah. It was quite rushed. Patient, NT*

Many patients recalled receiving written information from the genetic service prior to their appointment, but few found this helpful in their preparation to attend—indeed most people said they had not read it. In the following quote, a carer describes how additional information about what to expect in the appointment would have made for a smoother experience for his family.

*So the geneticist did a physical examination of the children?*

*Yeah and they got some strange man wanting to look at their flossies.*^2^*So ... you probably need to communicate that, a bit better … But if I had a bit of prior notice, then I could have worked them up beforehand and you know, this is what's going to happen when we go in there. Carer, NT*

#### Affordability and accessibility

The clinical genetic services included in this study were provided free of charge to patients, including the cost of any testing that was deemed clinically appropriate. However, patients were often not informed of this before the appointment. This was a considerable source of anxiety to some patients and may be a barrier to some patients attending. One parent describes how they had “heard that exome panels can cost like 4 and a half to 7 and a half thousand dollars” (Parent, NT) and feared they would have to pay for it if it was recommended. Although the services themselves were cost free, affordability remained a significant barrier for some participants because of other costs associated attending the appointment. Some participants reported that car parking, fuel costs and organising time off work were significant barriers to attending appointments.

Aboriginal and Torres Strait Islander patients are generally eligible for free transport to medical appointments, such as via the Patient Assistance Transport Scheme or their Aboriginal Community Controlled Health Services, however many participants were not aware of these services. Patients from regional and remote areas who had accessed Patient Assistance Transport Scheme (or similar) had varied experiences, which tended to hinge on initiative taken by the patient’s referring practitioner, that practitioner’s knowledge of the system and ability to advocate on their patient’s behalf. In most cases funding was only provided for the patient to attend, whereas some people were funded to travel with a support person, or dependent children. Availability of funding support to ensure that patients do not have to attend appointments alone is an issue in all areas of health. However, it is particularly problematic in relation to attending clinical genetic services where the appointment might be relevant to other members of a family group. Many patients felt that expanded outreach clinics that provided specialist services in regional and remote areas would have been beneficial in terms of logistics and continuity of care.

### During the appointment

#### Aboriginal support services

None of the participants had used or been offered the support of an Aboriginal Liaison Officer during their clinical genetics appointment, despite being these services being available within the hospitals where the clinics were held.

*[I was] not really culturally supported, I don't think anyone really took notice of my culture, being Aboriginal, or anything like that. No support was given to me as a result of being Aboriginal, it was literally my mum that was there for me. Patient, QLD*

*Having that Indigenous person next to you makes you feel more comfortable and confident to ask questions and talk, you know, speak more instead of just, yep, which is what a lot of Indigenous people do. They're just like, yep. Even though they don't understand them, they go, yep. Okay. Yep. [ … ] It almost relaxes the stresses down a bit so the important stuff can come out. Parent, NT*

A number of interviewees expressed surprise that a mainstream health service would take interest in their feeling culturally supported but agreed that this would have improved the experience they had when accessing the clinical genetics service. When asked about whether they would have taken up the opportunity to have an Aboriginal Liaison Officer present during their appointment, many participants indicated that they would. Others expressed that having genetic health professionals of Aboriginal and Torres Strait Islander heritage would make them feel more comfortable and supported in understanding the process of assessment and diagnosis for inherited conditions.

*I think would be really good, when you get your letter and that, to have an inclusive thing cause on that letter, doesn't ask, "Do you need an Aboriginal person to come with you?" It doesn't ask anything like that. Now that you talk about it, it would be good, but it never ever crosses my mind. I just go to my appointments. Parent, QLD*

*I would have loved that because there are a lot of health issues that affect Aboriginal-Torres Strait Islanders more so, or histories and their dynamics of family are a lot different as well. If I had the option of having an Aboriginal liaison officer with me, I would have said yes to it every single time. I've got eight kids, so you can imagine how many times I'm in hospital, and having that person there that understood their culture and how some things are different in their culture to Europeans, that would just be amazing. Parent, WA*

Lack of Aboriginal and Torres Strait Islander support meant that important issues from a socio-cultural perspective were not recognised or managed. Among Aboriginal and Torres Strait Islander women, gender may impact interactions between patient and practitioner based on cultural norms, lived experience and personal preference. A male guardian of female children also stated his preference for another woman to be present for physical examinations. Provision of culturally safe care should include awareness of how gender may act as a barrier to engagement more frequently among Aboriginal and Torres Strait Islander patients.

*So I have anxiety when it comes to speaking to men, so the doctor who we spoke to was a man, and it was harder to concentrate and be calm. Whereas, if it would have been a female, I would have been a lot more calmer. If it would have been an Indigenous person, I would have been a hell of a lot calmer. But understanding that Indigenous people have a problem with, culturally, a man and a woman …*

*Mm-hmm, talking?*

*Yeah. Talking and socializing and giving information to each other is sometimes a big no-no. Parent, NT*

*I knew that I was being listened to, because they answered the questions that I was asking. What was intimidating was the setting, and it being just me in a sterile room with this doctor. And a male doctor. Patient, QLD*

The reasons indicated for the preference for having an Aboriginal Liaison Officer present encompassed a number of themes, including having “another set of ears” (Patient, QLD) or who someone who “could break down or explain things along the way” (Parent, WA), alleviating concerns about gender, having someone to advocate on their behalf when delays or barriers were experienced, and specifically because they would feel more comfortable in a consultation if another Aboriginal person were there. Regardless of the motivation, or perceived “need”, this group of patients indicated that they wish to have their Aboriginality acknowledged in their interaction with the clinical genetics service.

#### Communication

Participants described feeling overwhelmed by the amount of information they had to take in during their appointments. Furthermore, they were candid about the fact that they had little prior knowledge of genetics in general. However, there were also a patients who felt that the breadth and depth of information provided to them was very appropriate and easy to understand. It should be noted that patients who felt the communication had been appropriate were most often those who had been diagnosed with a monogenic disorder, the implication being that these conditions have both a relatively simpler pattern of heritability and more direct relationship between genetic variant and phenotype.

Clinicians’ communication style during the appointments did not always support patients to develop an appropriate understanding of key concepts, such as the information that testing will provide and eligibility to access it, risk to the individual, family members and future children, the likelihood of shared aetiology with other conditions in the family and detection of benign variants or variants of unknown significance. Some patients were unable to understand the complex medical terms and concepts discussed in the consultation. This contributed to dissatisfaction and ongoing anxiety long after attending the service. As one mother aptly put it: *“This is my boy’s health! Talk straight to me!”* (Parent, NT). Another participant described “feel [ing] like an idiot” when she did not understand her practitioners. She responded by “smil[ing] and nod[ding], making notes [with the intention to] google that after and find out what it means” (Parent, WA).

The patient journey to and from clinical genetic services means that there are often limited opportunities to clarify understanding. The decision whether to undertake testing is usually finalised in the first appointment and results returned in a second, without further patient follow-up or discussion of results. This also means that patients may be left in doubt about appropriate next steps. The sentiment was also expressed that an additional follow up appointment would help them to better understand their results and “ask the right questions” about their implications (Patient, WA).

#### Inclusive and appropriate environments

Participants' confidence and ability to engage with their clinician was further limited by the physical and social environment of the services. Waiting and consulting rooms tended to be sterile and alienating with few welcoming signifiers for Aboriginal people. While primary health care services in Australia often include Aboriginal-themed posters, Aboriginal-specific public health information or an Aboriginal flag that serve to make Aboriginal patients feel more comfortable, these are not generally included in genetic health service environments.

The lack of representation of Aboriginal and Torres Strait Islander people within the services, be that as members of the workforce or in brochures, posters and information sheets, indicated to patients that genetic conditions are not something that Aboriginal people should be concerned about. A number of patients reported that there were very few Aboriginal and Torres Strait Islander people attending the services and that this contributed to their feeling of isolation.

*We got, "We've gotta keep our ears clean. We've gotta keep our hands clean." We've got all of those posters. There's nothing on genetics. We don't have anything on that and how to explain it. Parent, NT*

*To this day I still don't know if there is another Aboriginal person out there with what [son] has because I don't think they had that information. Parent, WA*

The nature of clinical genetics means that whole families were sometimes required to come in for consultations. Consequently, patients were often distracted by their family responsibilities with children “bouncing off walls” (Parent, NT) and were unable to fully engage in the consultation. One patient felt that the practitioner was also distracted by the children present and “couldn’t wait to get us out the door” (Parent, NT), which impacted on the quality of health information imparted in the consultation. Parents reported that small waiting rooms without facilities for entertaining children, or those that did not adequately accommodate mobility devices were another barrier to positive experiences of attending genetic health services.

### Post-appointment

#### Support: appointment outcomes and psychosocial needs

Most participants felt that there was insufficient support or opportunity for further discussion following their appointment. There were mixed experiences of receiving a report or letter detailing what was discussed in the appointment, although when this occurred it was generally found to be helpful. One parent appreciated the written information although felt they “still couldn’t really wrap [their] head around it” (Parent, QLD).

There was indication from participants that a follow-up call from the doctor or genetic counsellor would have been appreciated after they had had an opportunity to process the information they received and to think of any questions that were not answered during their consultation, or in the course of their own research afterwards.

*I think, yeah, a follow-up phone call within the next week would have been really helpful. Especially for me because I didn't take much during that meeting. Parent, NT*

*It was only afterward, and you're like, "Okay. What about, what about, what about?" Then, it's all too late by that point. Parent, QLD*

The need for support for the psychological and mental health sequelae of interactions with clinical genetic services was also clear.

*I felt lost. I had so many people [different doctors] to see and stuff, which is fair enough, but I suppose just to ask if I was all right and how do I think I'm going to be able to move forward doing this. Parent, QLD*

*Interviewer: And so the overall experience, what do you think was the most challenging?*

*Parent: The processing it all. Maybe another, like, check-up just to check on me sort of thing. Parent, QLD*

Parents of children with rare diseases described overwhelming feelings of isolation. Online communities (such as Facebook groups) were an important source of both support and information, however these were often found as a result of the parents’ own research, rather than on the suggestion of the practitioner or genetic counsellor. There was also a strong desire to form linkages with other Aboriginal families, in order to have their life experience, worldview and perceptions of disability that may differ from those of white parents validated. The lack of Aboriginal-specific support groups for most genetic conditions was an issue for some parents, such as one who “felt worse” after accessing a mainstream support group that felt alienating (Parent, WA).

Themes relating to post-appointment support are equally, if not more, relevant to patients and families for whom attending the genetics services does not achieve a definitive diagnosis. As this group continue on their diagnostic odyssey, feelings of helplessness, uncertainty and confusion are common, however among our participants, there were no examples of these patients receiving appropriate psychological support after attending genetic services. One parent described the distress she felt throughout her sons’ childhoods:

*I only did it [genetic testing] because I was just trying to really find out like I said, I don't know like, did I give them this condition because like, it's been really hard for me, for many, many years with no help from anyone, really. [ … ] I'm just confused, like, one minute, they said that it wasn't genetic—for years. I was just struggling right from when they were three and five, when they were diagnosed, and went through numerous schools like, so many, and just feeling so like, well, helpless, really, because I couldn't—I didn't know how to help them. Parent, QLD*

#### Planning for the future

A significant source of anxiety expressed by a number of parents related to being asked to return to the service with their children at some point in the future (such as “five years” or “when the children are adults”), without a definitive time frame being given, nor the ability to arrange a reminder call to arrange an appointment. One parent suggested she “thought waiting until they’re in their teens was a bit harsh.” (Parent, QLD).

A few patients who were diagnosed with a condition they were at risk of passing on to their children were told to return when they were considering starting a family. While discussing the reproductive implications of a diagnosis may not be of immediate concern, this gap in information was a significant burden, especially among young women who were pragmatic about the reality of unplanned pregnancies. One mother spoke about continued feelings of uncertainty and stress more than five years after attending the service indicating that she thought there was a real possibility one of her sons might have a child before they returned to the genetic service in their late teens to discuss the risk to their offspring (Parent, QLD). This was also the case for one young woman who was learned she was at increased risk of cancer in her early teens:

*Interviewer: Do you feel as though you needed more information about things, you know, to look at in the future?*

*Interviewee: Um, the only thing probably would be um, like pregnancy. We got told when we were there about um, coming back and talking to someone about when you’re wanting to try to have children. So, that was probably the only thing.*

## Discussion

**Fig. 1:**
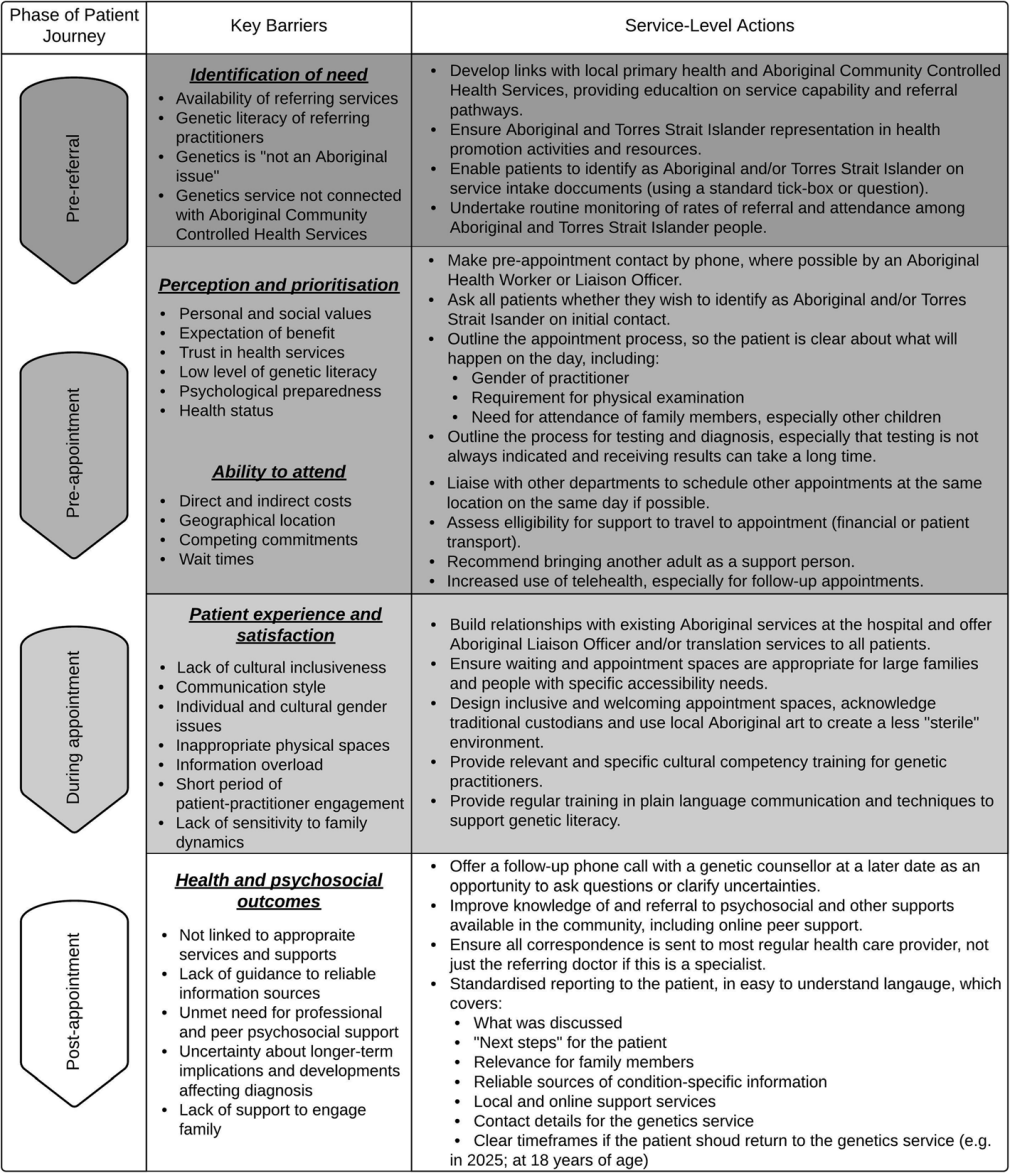
Barriers to accessing appropriate and culturally safe care exist at each phase of the patient journey to attending a clinical genetics service. Examples of key patient concerns raised by study participants which impacted their engagement are given for each phase and we offer corresponding recommendations to clinical genetics service providers for actions to address these

The results of this study highlight barriers to Aboriginal and Torres Strait Islander peoples’ participation in clinical genetic services throughout the patient journey. Addressing these issues is likely to improve the representation of Aboriginal and Torres Strait Islander people at these services, as well as benefit the broader patient population [39]. The current experiences of clinical genetics services anticipate considerations that will be necessary to support equity as genomic medicine is increasingly integrated into other areas of the health system. Figure 1 outlines changes to service provision that are able to be implemented within the current paradigm of genomic medicine that will improve access and outcomes for Aboriginal and Torres Strait Islander people who stand to benefit from clinical genetics services. Service providers are responsible for making proactive and immediate changes at the individual- and service-level to ensure that inequitably distributed benefits of genomic healthcare do not exacerbate the existing gap in health outcomes between Aboriginal and Torres Strait Islander people and other Australians.

Currently, there are no guidelines or policies for clinical genetic services that ‘recognise the collective cultural needs of Aboriginal and Torres Strait Islander people in relation to healthcare’ [6], although these exist in many other areas of the Australian health system. Our findings highlight the importance of developing such guidelines to support the services to ameliorate the under-representation of Aboriginal and Torres Strait Islander people. We suggest that there is unmet need in provision of practical and specific cultural safety training for genetic practitioners, which would empower and equip them to provide high quality care in what is often a brief clinical relationship. It is critical to the provision of greater cultural support that Aboriginal and Torres Strait Islander people are identified in clinical genetic service data [40] and that the risks associated with identification (e.g. racism) are mitigated [41]. Our findings demonstrate the importance of allowing Aboriginal and Torres Strait Islander patients the opportunity to identify themselves as such, so that improved support for individual and collective identity may be incorporated into their care and patients can access the services to which they are entitled. Improved identification of Aboriginal and Torres Strait Islander patients in clinical genetic records would have the additional advantage of enabling equity to be monitored. In addition, cultural safety training should encompass the specific support required pre-, during and post- appointment as outlined in Fig. 1. This will entail both improving the adequacy of service design and the clarity of communication.

The ability of clinical genetic services to deliver culturally safe care would be greatly enhanced by harnessing strengths of the existing Aboriginal and Torres Strait Islander health workforce. Establishing links with Aboriginal and Torres Strait Islander support services within the health settings where genetic services operate, as well as in the community, will further bolster the ability of clinical genetics services to provide culturally safe care and ensure that patients are able to access the full range the cultural, health and social support available. Recognising Aboriginal Community Controlled Health Services as a key point of contact with the health system for many Aboriginal and Torres Strait Islander people, and as a rich source of knowledge about the community they service, is also critical to improving continuity of care for patients with complex conditions and engaging new patients who stand to benefit.

Addressing barriers evident in the pre-appointment phase of the patient journey is critical to improving access to clinical genetic services for Aboriginal and Torres Strait Islander people. Firstly, difficulty in accessing appropriate services, including clinical genetics, is a common experience among people with rare conditions and people who are at-risk but pre-symptomatic, and is not unique to Aboriginal and Torres Strait Islander patients. However, some patients’ experiences of being dismissed when they requested a referral to a clinical genetics service does highlight the importance of improving genetic literacy and knowledge of referral pathways among primary care providers, including those practicing at Aboriginal Community Controlled Health Services.

Reflecting key themes from our findings, the suggested actions in Fig. 1 focus on improving the affordability and accessibility of services and ensuring the patient or family is aware of the purpose and process of the appointment before arriving. Pre-clinic contact appears to be a missed opportunity to encourage attendance and manage expectations, to ensure the patient has a good understanding of what will happen during the appointment and to discuss required support, both psychological and financial. Suggesting that patients bring another adult for support if they feel comfortable doing so is also likely to improve patient satisfaction following the appointment, particularly if it is indicated that the nature of the information that will be discussed could be new, complex and overwhelming, and that having a ‘second pair of ears’ is something many patients find useful. Normalising these responses to genetic information and appointments is likely to empower patients to ask more questions and seek alternative sources of information as suggested by the clinician [42], rather than turning to Dr. Google.

Improving patient preparation for appointments also relies on improved genetic literacy of referring practitioners, with a focus on clinical applications rather than biochemical mechanisms. Self-reported and/or objectively measured deficits in genetic literacy have been noted among specialist practitioners [43], nurses and midwives [44] and general practitioners [45] in Australia. We suggest that this requires stronger relationships between referring doctors in both primary and specialist care, as well as increasing the visibility of clinical genetic services and ongoing knowledge sharing. Our recommendations to improve affordability and accessibility focus on genetic services taking shared responsibility in linking patients with existing resources, such as Patient Assisted Transport Schemes. They also highlight the importance of considering how clinical genetic services can better engage with Aboriginal and Torres Strait Islander families. Together, these approaches have had demonstrable benefits in improving Aboriginal and Torres Strait representation in other areas of health [46].

Our findings suggest that many patients, although well aware of the health issue for which they had been referred, did not have a clear understanding of what clinical genetic services could contribute to addressing it. In terms of clinical interactions, it should not be assumed that patients understand the purpose of their referral or the nature of the services they have been referred to. This should be clearly discussed with the patient both prior to and in the appointment to make sure that there is a shared understanding of all the potential outcomes of genetic testing, in particular the likelihood and interpretation of uncertain findings [47].

During the appointment it is critical to create an environment where patients feel supported and are able to engage in a two-way dialogue with clinicians. This requires creating a culturally supportive physical environment where Aboriginal people see themselves represented, that considers the specific needs of children and families and acknowledges personal or community history of trauma in interactions with health services [48].

Lack of support following clinical genetic appointments was a significant barrier to the benefits of the service being realised for Aboriginal and Torres Strait Islander people. As clinical genetic services often work in a way that is disconnected from patients’ regular healthcare setting, patients sometimes do not receive directions in finding reliable information or appropriate support. Addressing this requires stronger linkages between the genetics service and a patient’s usual healthcare provider, not only the doctor who referred them (where these are different). This supports the imperative to build capacity in primary care, particularly Aboriginal Community Controlled Health Services, as well as to ensure that patients always receive a summary of relevant information directly. Post-appointment reports to doctors and patients should include information on reliable and appropriate sources of further information and support (both psychosocial, for example online groups for rare diseases, and practical, for example information about navigating the Australian National Disability Insurance Scheme with an undiagnosed condition). Living with a genetic condition, rare disease or undiagnosed condition is increasingly recognised to have a major impact on mental health for some patients and families [49]. It is important that clinical support encompasses all the implications of a genetic diagnosis. Similarly, clinicians must recognise that attending a clinical genetic service may be a single step in a long diagnostic odyssey for patients who do not receive a diagnosis [1]. These patients and families may require additional support.

### Limitations

As an exploratory study, and the first to investigate the perspectives of Aboriginal and Torres Strait Islander people on accessing clinical genetic services, our findings have some inherent limitations. Firstly, we only interviewed people who had attended the services, meaning that they had overcome barriers in the pre-appointment stage that prevent other Aboriginal and Torres Strait Islander people who stand to benefit from attending. Secondly, the lack of detailed demographic and clinical information about interviewed patients and/or their children does limit more nuanced understanding of how the needs of patients and families differ based on, for example, type of genetic condition, remoteness of residence, socio-economic status, etc.. Finally, the vast majority of interviewees were women, which is in line with previous findings that men attend genetic services at lower rates than women and are less likely to be the primary care giver for children. It will be important in the future to investigate the perceptions of Aboriginal men in a targeted way, so that they are equal recipients of the benefits of genomic medicine into the future.

## Conclusion

This research is the first study of Aboriginal and Torres Strait Islander patients’ experiences when interacting with clinical genetic services. Although the setting is the Australian health system, the issues raised are likely to apply to clinical genetic services globally. Central to improving the benefits of clinical genetic services to Indigenous peoples is creating systems to ameliorate information exchange and linkage between clinical genetic services and the rest of the health system [50, 51]. Achieving this requires two way learning with cultural knowledge from the Aboriginal Community Controlled Health sector and Aboriginal and Torres Strait Islander support services in the community. This approach will contribute to an integrated sense of collective identify in cultural safety training for clinical genetic practitioners. While genetic health practitioners must support genetic literacy within Aboriginal and Torres Strait Islander health services, it is also critical that clinical genetic services ensure that Aboriginal and Torres Strait Islander patients are able to access all the services they are entitled to by providing better support before, during and after their appointments, creating more welcoming environments and establishing pathways for ongoing communication to promote shared understanding between clinicians and patients [52]. Our paper highlights changes that can be made to improve access to clinical genetic services now. These are only one part of the systemic changes required to make access to genomic medicine equitable.

## Supplementary Information

**Additional file 1.** Interview Guide.

**Additional file 2.** Final Coding Framework.

## Data Availability

The datasets generated during and/or analysed during the current study are not publicly available due to the personally identifying nature of the interviews, which are not able to be completely de-identified as a result of the rare conditions and remote locations of some of the participants.

## Footnotes

1 The most recent version of this document was published in 2013. Currently, the HGSA Policies webpage states: “The previously published Clinical Genetics Services Standards Framework has been withdrawn pending a complete review which is under consideration”.

2 Australian slang – bottom or genitalia.
