# Supplementary material for: “This is my boy’s health! Talk straight to me!” perspectives on accessible and culturally safe care among Aboriginal and Torres Strait Islander patients of clinical genetics services
Source: Int J Equity Health. 2021 Apr 17;20:103. doi: 10.1186/s12939-021-01443-0 (PMC8052687; doi:10.1186/s12939-021-01443-0)
Supplement: Supplementary file 1 — Additional file 1. Interview Guide. [file 12939_2021_1443_MOESM1_ESM.pdf]

Supplementary Material: Interview Guide

For Consumers / Guardians

(patients who have attended one of the clinical genetic service providers involved in project)

Consumers that are participating in patient journey mapping will be given the opportunity to share all aspects of their experiences in accessing clinical genetic services. Therefore, the flow of the interview will be largely dictated by the consumer. The interviewer will use the following prompts to guide the interview if necessary, ideally in an order that follows the chronological order of the patient journey.

The interviewer will begin by discussing the purpose of the research study, talking through the 'plain language statement' and ensuring they have given informed consent to participate. They will then gain verbal consent to begin audio recording.

- ➔ So <name>, where do you live?
- ➔ Do you identify with specific communities and/or language groups?
- ➔ When did you last visit <clinical genetic service provider>? Was it your first visit?
- ➔ Can you explain to me **why** you were there?
- ➔ Do you know if any of your blood relatives have this same "*thing*"? *(researcher to add whatever was explained as the reason for visit)* If yes – Does that worry you?
- ➔ Did you get answers for any questions you had going into your visit to the genetic service provider? (were your expectations met?)
- ➔ What do you think about when I say "genetics"? For example do you think of things that are passed through blood lines? Or family lines? How would you describe it?
- ➔ Could you tell me your story about going to <clinical genetic service provider> including before and after the visit?

After a narrative account has been given, the interviewer, together with the participant, will add make a pictorial account using cards that depict the different locations of a patient journey eg. home, clinic, travel etc. For each stage of the journey, the researcher will ask the participant how they felt at that stage. If not already covered by the narrative provided by the participant, the interviewer will use the following prompts.

Pre-Visit

- ➔ Who first told you that you need to go to visit <clinical genetic service provider>?
- ➔ How did you feel when you they told that you needed to visit <clinical genetic service provider>? Did you have any concerns? Worries?
- ➔ What were the reactions of your family and community? Was there anyone you could talk to about it? Did you feel supported?
- ➔ Did you understand why you were told you that you need to go to visit <clinical genetic service provider>?
- ➔ Were you told what would happen during the visit to <clinical genetic service provider>?
- ➔ Did you receive any information about 'genetics' (*or the condition*) before your visit to <clinical genetic service provider>? If yes – Was it understandable? Useful? Why or why not?

- ➔ Did you feel you were well supported by your local clinic? Did they offer to help with any services such as transport?
- ➔ Did you use any Aboriginal Community Controlled Health Services (ACCHSs) in getting ready to visit <clinical genetic service provider>? If yes – Did you find them helpful? In what way?
- ➔ Do you remember how much time passed between first going to the clinic, being told you need to visit <clinical genetic service provider>, getting an appointment, and your visit to <clinical genetic service provider>?

Visit

- ➔ Did you use any Aboriginal Services during your visit to <clinical genetic service provider>? If yes- did you find them helpful? In what way?
- ➔ Did you need to find accommodation in the city where the <clinical genetic service provider> appointment> was? ? If yes – can you tell me about this?
- ➔ How did you get to <clinical genetic service provider> appointment>? Did you receive any help for example transport or financial?
- ➔ Did you received clear instructions in how to find the <clinical genetic service provider> appointment place? Did you have any trouble finding it?
- ➔ Did anyone come with you to the <clinical genetic service provider> appointment>? If yes- who? How was that? Did it make it easier having them there? Or would prefer to go to the appointment alone?
- ➔ How were you feeling when going into your appointment at the <clinical genetic service provider>?
- ➔ Did the doctor explain things in a way that made it easy for you to understand?
- ➔ When you didn't understand something, did you feel comfortable asking questions?
- ➔ What helped/hindered you feeling listened to at the appointment?
- ➔ What things about the visit helped/hindered you feeling supported culturally?
- ➔ Did you receive any information at your visit? Was it understandable or useful? Why or why not?
- ➔ How did you feel straight after the appointment was finished?
- ➔ What issues, if any, would you have liked more information about **in** the appointment?
- ➔ What issues, if any, would you have liked more information about **after** the appointment?
- ➔ Is there anything that you would have liked to be different about the consultations? If so what?
- ➔ Would have you liked it better if the appointment was at a ACCHSs instead? If yes – why? If no- why not?

Post-Visit

- ➔ How did you get back to your community after the appointment? How was that?
- ➔ Did you tell the staff at your local clinic about your visit?
- ➔ Did you feel supported by your local clinic? Did they offer you any services? If yes - what did they offer? If no - what would have you liked them to offer?
- ➔ Did you use any ACCHSs during when you came back home? Could you tell me more about that?
- ➔ Do you think that services you were provided after your visit to <clinical genetic service provider> were different from what you were offered before you went?

Overall

- ➔ What was the most challenging or negative (worse) thing about your experiences in accessing (going to) the genetic services?
- ➔ What was the most positive or best thing about your experiences in accessing (going to) the genetic services?
- ➔ Is there anything else that is important to you that we haven't talked about? *example; spirituality, family, access, communication*
