# Supplementary material for: “This is my boy’s health! Talk straight to me!” perspectives on accessible and culturally safe care among Aboriginal and Torres Strait Islander patients of clinical genetics services
Source: Int J Equity Health. 2021 Apr 17;20:103. doi: 10.1186/s12939-021-01443-0 (PMC8052687; doi:10.1186/s12939-021-01443-0)
Supplement: Supplementary file 2 — Additional file 2. Final Coding Framework. [file 12939_2021_1443_MOESM2_ESM.docx]

Supplementary Material: Final Coding Framework

| Theme (stage of the patient journey) | Nodes |
| --- | --- |
| Being referred to clinical genetics service | - Prior genetic literacy - What is this appointment for? - Expectations of outcomes - Problems obtaining referral   - Referring practitioner misinformation – cost   - Referring practitioner misinformation – risk   - Not offered |
| Preparation for appointment | - Contact with service before appointment - Motivation to attend - Wait times   - Worry   - Forgot   - “Is this important?” - Referral during pregnancy - Other health priorities - Never heard of genetics |
| Getting to (and from) the appointment | - Logistical issues   - Travel time/distance   - Cost of hospital parking   - Patient Assisted Transport Scheme   - ACCHS arranged travel   - Financial support? - Competing demands on day of appointment   - Work   - Care giving |
| In the appointment | - Environment   - Small consult rooms/waiting areas   - Sterile   - Children who “can’t sit still” - Physical examinations/taking samples   - Not warned   - Not comfortable with male doctor   - Photos - Communication   - Information overload   - Medical jargon   - Diagrams helpful   - Confident to ask questions - Aboriginal Liaison Officers - Recognition of Aboriginality? |
| Post-appointment (short term) | - Communication with referring doctor   - Discontinuity of care - Follow-up - Own research - Results   - Waiting periods     - Impact on other health/reproductive choices   - “Now what?”   - Variants of Unknown Significance/”no results”   - Talking to family - Patient reports/letters |
| Post-appointment (long-term) | - Benefits of attending   - End of diagnostic odyssey   - Changes to treatment/management   - Preventative measures   - Reproductive planning   - National Disability Insurance Scheme   - Alleviation of guilt   - Explaining to family - “No answers” - Long time frame reviews - Confusion about family eligibility for testing - What is and isn’t ‘genetic’ – post-appointment genetic literacy |
| Theme (crosscutting) |  |
| Support | - ACCHSs - Family - Facebook groups - Parents groups |
| Culture | - Language - Gender - Relationships - Concern for “people from community” |
| Family dynamics | - No family history - Fragmented/no communication - Refusal to provide samples (other parent) - Looking after each other/our grandchildren - “Do they have access?” |
| Experiences of racism in healthcare | - General perceptions and experiences - Directly related to the health issue discussed |
| Perception of need (for appointment/testing) |  |
